# Microbial community composition explains soil respiration responses to changing carbon inputs along an Andes-to-Amazon elevation gradient

**DOI:** 10.1111/1365-2745.12247

**Published:** 2014-05-19

**Authors:** Jeanette Whitaker, Nicholas Ostle, Andrew T Nottingham, Adan Ccahuana, Norma Salinas, Richard D Bardgett, Patrick Meir, Niall P McNamara, Amy Austin

**Affiliations:** 1 Centre for Ecology and Hydrology, Lancaster Environment CentreLibrary Avenue, Lancaster, LA1 4AP, UK; 2 Lancaster Environment Centre, Lancaster UniversityLancaster, LA1 4YQ, UK; 3 School of Geosciences, University of EdinburghThe King's Buildings, West Mains Road, Edinburgh, EH9 3JW, UK; 4 Facultad de Ciencias Biologicas, Universidad Nacional de San Antonio Abad delCusco, Avenida de la Cultura 733, Cusco, Peru; 5 Seccion Química, Pontificia Universidad Católica del PeruAv. Universitaria 1801, San Miguel, Lima 32, Peru; 6 Faculty of Life Sciences, The University of ManchesterMichael Smith Building, Oxford Road, Manchester, M13 9PT, UK; 7 Research School of Biology, The Australian National UniversityCanberra, ACT 0200, Australia

**Keywords:** bacterial, carbon substrates, decomposition, ecosystem function, fungal, microbial community composition, montane cloud forest, plant–soil (below-ground) interactions

## Abstract

1. The Andes are predicted to warm by 3–5 °C this century with the potential to alter the processes regulating carbon (C) cycling in these tropical forest soils. This rapid warming is expected to stimulate soil microbial respiration and change plant species distributions, thereby affecting the quantity and quality of C inputs to the soil and influencing the quantity of soil-derived CO_2_ released to the atmosphere.

2. We studied tropical lowland, premontane and montane forest soils taken from along a 3200-m elevation gradient located in south-east Andean Peru. We determined how soil microbial communities and abiotic soil properties differed with elevation. We then examined how these differences in microbial composition and soil abiotic properties affected soil C-cycling processes, by amending soils with C substrates varying in complexity and measuring soil heterotrophic respiration (R_H_).

3. Our results show that there were consistent patterns of change in soil biotic and abiotic properties with elevation. Microbial biomass and the abundance of fungi relative to bacteria increased significantly with elevation, and these differences in microbial community composition were strongly correlated with greater soil C content and C:N (nitrogen) ratios. We also found that R_H_ increased with added C substrate quality and quantity and was positively related to microbial biomass and fungal abundance.

4. Statistical modelling revealed that R_H_ responses to changing C inputs were best predicted by soil pH and microbial community composition, with the abundance of fungi relative to bacteria, and abundance of gram-positive relative to gram-negative bacteria explaining much of the model variance.

5. *Synthesis*. Our results show that the relative abundance of microbial functional groups is an important determinant of R_H_ responses to changing C inputs along an extensive tropical elevation gradient in Andean Peru. Although we do not make an experimental test of the effects of climate change on soil, these results challenge the assumption that different soil microbial communities will be ‘functionally equivalent’ as climate change progresses, and they emphasize the need for better ecological metrics of soil microbial communities to help predict C cycle responses to climate change in tropical biomes.

## Introduction

Tropical forests make a substantial contribution to the global carbon (C) cycle as they are highly productive, hold 30% of the Earth's soil C stock and exhibit the highest rates of soil respiration of any terrestrial ecosystem (Jobbagy & Jackson 2000; Bond-Lamberty, Wang & Gower 2004). In the Andes, tropical forests extend from the lowlands to upwards of 3000 m asl (above sea level), and they are recognized as one of the most biologically diverse regions of the planet (Myers *et al*. 2000; Malhi *et al*. 2010). In the montane cloud forests that dominate the middle-upper elevations of the Andes, wet and relatively cool conditions prevail (Grubb 1977); this results in suppressed rates of decomposition and accumulation of extensive stocks of soil organic matter (Zimmermann *et al*. 2009). There is, however, growing concern that atmospheric warming as a result of climate change (IPCC 2013) could lead to significant changes in soil C cycling due to direct effects on microbial breakdown of soil organic matter, soil respiration and greenhouse gas feedbacks to the atmosphere (Bardgett, Freeman & Ostle 2008; Craine, Fierer & McLauchlan 2010; Schindlbacher *et al*. 2011).

In addition to direct temperature and precipitation effects, climate can indirectly affect soil respiration by influencing plant community productivity and structure, which in turn determines the quantity and quality of C inputs entering the soil (Engelbrecht *et al*. 2007; Feeley *et al*. 2011; Garcia-Palacios *et al*. 2012). Sources of C inputs, including plant litter and rhizodeposition, act as substrates that are mineralized to CO_2_ by the soil microbial community. Changes in the quality of these C inputs occur as a result of differences in plant species tissue chemistry that affect the nutrient stoichiometry of their litter and rhizosphere inputs (Hättenschwiler *et al*. 2008). Consequently, changes in plant community composition can lead to shifts in the proportion of recalcitrant (e.g. lignins) and accessible (e.g. non-structural carbohydrates) forms of C in plant-derived substrates, altering litter quality (Hattenschwiler & Jorgensen 2010). These indirect, plant-mediated effects of climate change on microbial activity and soil respiration are poorly understood and represent a significant knowledge gap in determining the response of terrestrial C cycling to future climate change (Bragazza *et al*. 2013; Ward *et al*. 2013).

Soil abiotic and biotic properties are also recognized as strong determinants of soil C cycling across ecosystem types (Smith *et al*. 2008; Chapin *et al*. 2009). In C-rich soils, found in peatlands and forests, the nutrient characteristics of the organic matter can be a significant predictor of decomposition and soil heterotrophic respiration (R_H_; Bragazza *et al*. 2006; Fisher *et al*. 2013). At the same time, dominant and abundant taxa of soil bacteria and fungi have differing capacities to degrade available and complex forms of plant-derived C and N resources (Berg & Laskowski 2006; Waring, Averill & Hawkes 2013). For example, fungi are known to have broader enzymatic capabilities (McGuire *et al*. 2010), slower biomass turnover rates (Rousk & Bååth 2011) and potentially greater C use efficiency (Six *et al*. 2006) than bacteria. This suggests that differences in the relative abundance of fungi and bacteria, or their constituent groups, could potentially affect soil C cycling, and in particular R_H_ responses to changing plant-C inputs (Bailey, Smith & Bolton 2002; Waring, Averill & Hawkes 2013). The effects of differences in soil microbial community composition on C-cycling processes, however, remain poorly quantified (Schimel & Schaeffer 2012). Comparative studies of C substrate decomposition by soil microbial communities are generally only conducted on one or two soils (Brant, Sulzman & Myrold 2006; Hanson *et al*. 2008; Steinweg *et al*. 2008; Rinnan & Bååth 2009; Eilers *et al*. 2010), and few have considered tropical forest soils (Cleveland *et al*. 2007; Leff *et al*. 2012). This lack of evidence has led to an assumption that different soil microbial communities are ‘functionally equivalent’ with decomposition principally controlled by abiotic parameters, primarily temperature and moisture (Strickland *et al*. 2009). There are clearly significant gaps in understanding regarding the role of soil abiotic versus biotic properties as regulators of C cycle responses to changes in organic matter inputs, particularly in tropical forests.

In this study, we investigated how differences in soil properties, including microbial community composition, determine the response of R_H_ to changes in C inputs in tropical forest soils. For this, we measured abiotic and biotic properties and R_H_ responses in soils from 13 tropical forest sites located along an elevation gradient ranging from 194 to 3400 m asl in south-east Peru (Girardin *et al*. 2010; Malhi *et al*. 2010). The vegetation along this gradient ranges from lowland tropical forest to montane cloud forest, corresponding with changes in climate, underlying geology and topography (Table1). In these forests, total soil C stocks and the relative proportion of labile C in organic topsoils is known to increase significantly with elevation (Zimmermann *et al*. 2012). In addition, net primary productivity and nitrogen (N) availability broadly decrease with elevation as phosphorus (P) availability tends to increase (Tanner, Vitousek & Cuevas 1998; van de Weg *et al*. 2009; Fisher *et al*. 2013). We determined the response of R_H_ to changes in the quantity and form of C inputs, as a proxy for changes in litter inputs, and hypothesized that (i) along the gradient changes in soil microbial functional groups correspond with increases in soil organic matter C and N content; (ii) the magnitude of R_H_ responses to C inputs increase with their C quality and quantity, with labile substrates being mineralized most rapidly in high-elevation soils; and (iii) the relative abundance of microbial functional groups (fungi and bacteria, gram-negative and gram-positive bacteria) is an important determinant of R_H_ responses to C inputs, in soils varying in abiotic properties. To test hypothesis i, we measured a range of soil abiotic and biotic properties on samples taken from 13 locations along the elevation gradient. Two controlled substrate addition assays were then conducted (experiments 1 and 2) to address hypotheses ii and iii, respectively, with a statistical modelling approach (Experiment 2) used to examine the relationships between microbial and abiotic metrics as determinants of R_H_ responses.

**Table 1:** Summary of site characteristics along the elevation gradient (Aragao *et al*. 2009; Girardin *et al*. 2010; Quesada *et al*. 2010; Clark *et al*. 2013; Gurdak *et al*. 2014; Asner *et al*. 2014)

Site name	Site code	Elevation (m asl)	Lat	Long	Mean annual temp (°C)	Annual precipitation (mm year^−1^)	Organic layer (cm)	Aspect (deg)	Slope (deg)	Parent material	Soil classification
Explorer's Inn plot 4 (TP4)	TAM-06	194	−12.839	−69.296	26.4	2730	0.7	169.4	4.0*	Holocene alluvial terrace	Haplic Alisol
Explorer's Inn plot 3 (TP3)	TAM-05	210	−12.830	−69.271	26.4	3199	2.5	186.2	6.0†	Pleistocene alluvial terrace	Haplic Cambisol
Villa Carmen	VC	1000	−12.866	−71.401	20.7 ± 0.02	3087	3.6	na	na	na	na
San Pedro 2	SPD-2	1500	−13.049	−71.537	17.4 ± 1.5	2631	16.0	143.5	22.7†	Plutonic intrusion (granite)	Cambisol
San Pedro 1	SPD-1	1750	−13.047	−71.543	15.8 ± 1.3	2631	9.6	141.9	40.1*	Plutonic intrusion (granite)	Cambisol
Trocha Union 8	TRU-08	1850	−13.071	−71.555	16.0 ± 1.3	2472	15.6	137.0	41.8*	Plutonic intrusion (granite)	Cambisol
Trocha Union 7	TRU-07	2020	−13.074	−71.559	14.9 ± 1.1	1827	16.8	na	18.0‡	Palaeozoic shales-slates (granite)	Cambisol
Trocha Union 5	TRU-05	2520	−13.094	−71.574	12.1 ± 1.0	na	13.6	na	na	Palaeozoic shales-slates	na
Trocha Union 4	TRU-04	2720	−13.107	−71.589	11.1 ± 1.0	2318	21.4	189.8	21.0†	Palaeozoic shales-slates	Umbrisol
Trocha Union 3	TRU-03	3020	−13.109	−71.600	9.5 ± 1.0	1776	17.2	129.3	12.0‡	Palaeozoic shales-slates	Umbrisol
Wayqecha	WAY-01	3025	−13.190	−71.587	11.1 ± 1.2	1706	22.8	na	18.2†	Palaeozoic shales-slates	Umbrisol
Trocha Union 2	TRU-02	3200	−13.111	−71.604	8.9 ± 1.0	na	11.8	na	na	Palaeozoic shales-slates	Umbrisol
Trocha Union 1	TRU-01	3400	−13.114	−71.607	7.7 ± 1.1	2555	14.0	144.3	34.3‡	Palaeozoic shales-slates	Umbrisol

* Asner *et al*. (2014) 25-ha plot.

† Gurdak *et al*. (2014) 1-ha plot.

‡ Huasco (unpublished) 1-ha plot.

na, data not available.

## Materials and methods

### Study Site and Field Sampling

Soils were sampled in December 2010 from 13 sites along a tropical elevation gradient located on the east flank of the Peruvian Andes, with site elevations ranging from 194 to 3400 m asl. The transect from 1000 to 3400 m asl is 35 km in length and is sited predominantly on Palaeozoic (∼450 Ma) meta-sedimentary mudstones (∼80%), with plutonic intrusions (granite) underlying the sites between 1500 and 2020 m asl (Carlotto *et al*. 1996; Clark *et al*. 2013). The lowland sites at Tambopata are 230 km further down the valley from the 1000 m site. The soil at the 194 m lowland site is an alisol on a fertile Holocene alluvial terrace close to the river, whilst the soil at the 210 m site is on a relatively infertile Pleistocene cambisol (Aragao *et al*. 2009; Quesada *et al*. 2010). Soils from 1000 to 2020 m are also cambisols, whilst those above 2520 m are umbrisols (Table1). All the sites have continuous forest cover ranging from lowland Amazonian rain forest to upper montane cloud forest. Mean annual temperature decreases with increasing elevation along this transect, from 26 to 6 °C, whilst annual precipitation ranges from 1700 to 3087 mm year^−1^, peaking at 1000 m asl near the base of the mountains, then decreasing with elevation (Table1). Evidence to date indicates that these soils are rarely moisture limited over the seasonal cycle partly due to limited evapotranspiration and fog deposition within the cloud immersion zone (1500–3400 m asl; van de Weg *et al*. 2009; Zimmermann *et al*. 2010). Climate and environmental characteristics of the sites are described in Table1 with more detailed descriptions published elsewhere (Girardin *et al*. 2010; Quesada *et al*. 2010; Asner *et al*. 2014). At each site, soils were sampled from five subplots within established 1-ha plots. For each subplot, soil was removed from a 40 × 40 cm area to 10 cm depth. Soils were sealed in plastic bags, immediately transported to the laboratory and stored at 4 °C for 6 weeks until used for experimentation and analysis.

### Soil Abiotic and Biotic Properties

A range of analyses was conducted on soil samples taken from all subplots, to test whether changes in microbial functional groups along the gradient correspond with differences in soil abiotic properties (hypothesis 1). Soils were homogenized thoroughly by hand, and large stones and woody debris were removed. Analyses included pH (soil:H_2_O, 1:2.5 w:v), gravimetric moisture content (dried for 24 h at 105 °C) and total C and N content of dried, ground soil samples (approx. 100 mg) analysed using a TruSpec CN Elemental Determinator (LECO, St Joseph, Michigan, USA). Bulk density was analysed following the method of Emmett *et al*. (2008). Maximum water holding capacity (WHC) was calculated on composite soil samples for each elevation (composite of five subplots per site) as the amount of water remaining in the soil after being saturated and left to drain for 12 h in a fully humid airspace (Ohlinger 1995).

Soil microbial biomass C and N was measured by fumigation extraction (Brookes *et al*. 1985; Vance, Brookes & Jenkinson 1987) with resultant filtrates analysed for extractable microbial C using a Shimadzu 5000A TOC analyser (Shimadzu, Milton Keynes, UK) and for extractable microbial N (Ross 1992) by colorimetry on a continuous flow stream autoanalyser (Bran and Luebbe, Northampton, UK), as described in Ward *et al*. (2007). Extractable microbial biomass C and N was calculated from the difference between non-fumigated and fumigated samples and corrected for extraction efficiency using published conversion factors (Brookes *et al*. 1985; Vance, Brookes & Jenkinson 1987; Sparling *et al*. 1990).

Microbial community composition was determined using phospholipid fatty acid (PLFA) analysis, as described by Bardgett, Hobbs and Frostegard (1996). Phospholipids were extracted from 1.5 g soil fresh weight and analysed using an Agilent 6890 Gas Chromatograph. Gram-positive bacteria were identified by the terminal and mid-chain branched fatty acids (15:0i, 15:0a, 16:0i, 17:0i, 17:0a), and cyclopropyl saturated and monosaturated fatty acids (16:1ω7, 7,cy-17:0, 18:1ω7, 7,8cy-19:0) were considered indicative of gram-negative bacteria (Rinnan & Bååth 2009). The fatty acids 18:2ω6,9 and 18:1ω9 were considered to represent saprotrophic and ectomycorrhizal fungi (Kaiser *et al*. 2010; De Deyn *et al*. 2011). Total PLFA concentration was calculated from all identified PLFAs (15:0, 14:0, 16:1, 16:1ω5, 16:0, 17:1ω8, 7Me-17:0, br17:0, br18:0, 18:1ω5, 18:0, 19:1; plus those listed above). The ratios of fungal to bacterial (F:B) PLFA and gram-positive to gram-negative (GP:GN) PLFA were taken to represent the relative abundance metrics of these groups. To compare the relative abundance of fungi and bacteria on a biomass basis, the ratio of fungal biomass C to bacterial biomass C (F:B biomass C) was calculated by averaging empirically determined conversion factors (Keinanen *et al*. 2002; Bouillon *et al*. 2004; Klamer & Bååth 2004; Joergensen & Wichern 2008; Bezemer *et al*. 2010; Waring, Averill & Hawkes 2013).

### Experiment 1: Effects of C Quantity and Complexity on Soil Heterotrophic Respiration

Soils sampled from four plots situated at 210, 1500, 1750 and 3025 m asl were used to investigate the relationship between R_H_, increasing C inputs and C complexity (hypothesis 2). Subplot soil samples (5) for each location were combined, homogenized to form one sample and equilibrated for 7 days at 20 °C. Moisture content was adjusted to 80% of maximum WHC, and aliquots (6 g fwt) of each soil were placed in 175-ml Wheaton bottles. Soil from each elevation was amended with nine substrates at a range of concentrations (three replicates). Ecologically relevant substrates were selected to represent a range of chemical recalcitrance varying from labile, soluble compounds to complex, insoluble organic polymers. We classified these as simple, intermediate or complex based on these characteristics (Table2). The substrates glucose, xylose, cellobiose, glycine, n-acetyl glucosamine and vanillin (Sigma-Aldrich, Gillingham, UK) were prepared in serial dilution in sterile deionized water. Non-soluble substrates lignin (Sigma-Aldrich), hemicellulose and cellulose (IsoLife bv, Wageningen, the Netherlands) were diluted into suspension, sonicated for 10 min and vortexed for 5 s prior to pipetting and mixing into the soil. Dilutions were prepared so that each substrate was added to soil in 1 mL deionized water per incubation. Final concentrations of substrates added to soil were 0, 0.002, 0.02, 0.2, 2 mg C g^−1^ soil fwt for soluble substrates and 0, 0.002, 0.02, 0.2, 0.8 mg C g^−1^ soil fwt for non-soluble substrates. Following substrate addition, compressed air was used to flush the headspace for 1 min to achieve a standard starting atmosphere for each incubation. Bottles were then sealed with butyl rubber stoppers and aluminium crimp caps, over-pressurized by injecting 10 mL of compressed air, to allow for subsequent headspace sampling, and were incubated for 7 days at 20 °C in the dark. The headspace of each bottle was sampled at 24, 48 and 168 h by taking a 5-mL sample with an air-tight syringe and injecting it into a 3.5-mL exetainer vial (Labco, Lampeter, UK). Headspace CO_2_ samples were analysed on a PerkinElmer Autosystem GC fitted with a flame ionization detector containing a methanizer (Case *et al*. 2012). Results were calibrated against certified gas standards (BOC Ltd., Guildford, UK) and converted to a total CO_2_ flux reported as CO_2_-C (μg g soil dwt^−1^ day^−1^), in accordance with methods detailed in Holland *et al*. (1999). Basal respiration (BR) was defined as the measured CO_2_ flux in the absence of substrate addition. Substrate-induced respiration (SIR) was defined as the measured CO_2_ flux in the presence of added C substrate. As the soils varied significantly in their BR, the actual increase in CO_2_ emissions from soil following C additions was calculated by subtracting BR from SIR, and hereafter referred to as ‘additional CO_2_ flux’.

**Table 2:** Carbon substrate characteristics and biogeochemical relevance

Substrate	Complexity	Classification	Ecological relevance
Glucose	Simple	Monosaccharide	Product of decomposition processes
Xylose	Simple	Monosaccharide	Main building block for hemicellulose
Cellobiose	Simple	Disaccharide	Produced during hydrolysis of cellulose
Glycine	Simple	Amino acid	Root exudate, N source
N-acetyl glucosamine	Simple	Amino sugar	Product of chitin degradation
Vanillin	Intermediate	Benzaldehyde	Product of lignin depolymerization
Hemicellulose	Complex	Polysaccharide	Constituent of plant cell walls
Cellulose	Complex	Polysaccharide	Constituent of plant cell walls
Lignin	Complex	Complex organic polymer	Constituent of plant cell walls

### Experiment 2: Microbial Community Composition and R_H_ Response to C Inputs

To investigate the influence of the relative abundance of microbial functional groups in determining R_H_ responses to C inputs (hypothesis 3), soils from 11 locations along the gradient were incubated with four C substrates. Substrates were selected with different structural complexity to mimic plant-C inputs: two simple compounds (xylose and glycine), one intermediate (vanillin) and one complex compound (hemicellulose). Soils were incubated for 7 days at 20 °C in the dark with four C substrates at a dose rate of 0.2 mg C g fwt^−1^ soil. This concentration was selected based on earlier results in order to add sufficient C to increase R_H_ without inducing a significant increase in microbial biomass. Subplot soil samples (5) for each elevation were treated as individual replicates, and all soils were adjusted to 80% of maximum WHC and incubated with one of the substrates or a control treatment (sterile deionized water). Experimental set-up and sample analyses were described in Experiment 1, except 9 g fwt soil was used in each incubation bottle and headspace CO_2_ samples were taken at 48 and 168 h. The effects of substrate addition on the CO_2_ flux were calculated as additional CO_2_ flux (SIR-BR) as described earlier.

### Statistical Analysis

All statistical analyses were conducted using the statistical package R, version 2.14.0 (R Development Core Team 2008). Data were checked for normality and homogeneity of variance and transformed where necessary. Data from the analytical survey were analysed using linear regression (Sigmaplot v. 12.0, Systat Software Inc., Hounslow, UK) to determine how biotic and abiotic soil properties changed with elevation (hypothesis 1). Data from Experiment 1 were plotted and compared visually to identify how R_H_ responses varied in soils from four elevations, in response to increasing concentrations of C inputs of varied complexity (hypothesis 2).

To address hypothesis 3, data from Experiment 2 were firstly used to test whether R_H_ responses to C inputs differed significantly between 11 soils from along the gradient. Main and interactive effects of substrate and soil on additional CO_2_ fluxes were analysed by two-way analysis of variance (anova), with pairwise comparisons of interactive effects conducted using Tukey's HSD *post hoc* tests. To detect significant interactions, one-way anova with *post hoc* Tukey's HSD tests was conducted on soil and substrate data subsets. Following these analyses, we investigated how the relative abundance of microbial functional groups improved predictions of R_H_ responses to C inputs using linear mixed effects (LME) models. LME models of R_H_ were constructed for each substrate individually (including control treatment) and for all substrates combined. For each model, soil and substrate were included as random effects and soil abiotic and biotic properties specified as fixed effects. The measures of microbial community composition comprised total PLFA, bacterial PLFA, fungal PLFA, F:B PLFA and GP:GN PLFA. Additional abiotic and biotic properties included microbial biomass C and N, soil pH, soil C and N content, and soil C:N ratio. Models were refined and validated following the guidance provided in Zuur *et al*. (2010): all parameters were included in the initial model with non-significant terms removed manually in a systematic, stepwise process to achieve the best goodness-of-fit with fewest factors, assessed by selecting the model with the lowest Akaike Information Criterion (AIC). If removal of a non-significant term increased the AIC value, the term was retained in the refined model. Once the final models were reached for each of the substrate treatments, we fitted a linear model, removing random effects, in order to assess the significance of each term in the model. The adjusted *R*^2^ value of the fitted model was calculated and compared with the adjusted *R*^2^ of models fitted with each parameter removed in turn. The relative contribution of each parameter in explaining the variance of the model was then calculated as a percentage of the total variance explained.

## Results

### Soil Abiotic and Biotic Properties along the Elevation Gradient

**Figure 1:**
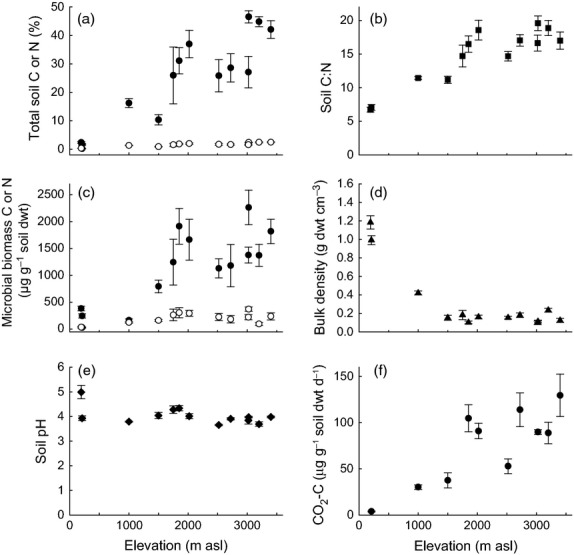
Soil chemical and physical properties along the elevation gradient (a) total C (●) and N (○) (%), (b) soil C:N ratio, (c) Microbial biomass C (●) and N (○) (μg g^−1^ soil dwt), (d) bulk density (g dwt cm^−3^), (e) soil pH and (f) basal respiration under standardized laboratory conditions (20 °C, 80% WHC). Data represent mean ± SE (*n* = 5). Statistical analysis is presented in Table3.

Soil abiotic properties in the surface soil (0–10 cm) varied significantly with elevation (Fig.1a–e and Table3). Total soil C and N and the soil C:N ratio increased significantly with increasing elevation (*P *<* *0.001; *R*^2^ = 0.538, 0.528, 0.617, respectively), whilst soil pH (*P *=* *0.008, *R*^2^ = 0.193) and bulk density (*P *<* *0.001, *R*^2^ = 0.368) decreased significantly with elevation (Table3). Total C increased from 1.7 % at 210 m to a maximum of 46.5 % at 3025 m, whilst the C:N ratio ranged from 6.7 at 210 m to 19.6 at 3025 m (Fig.1a,b). Soil pH was highest at the lowest elevation site (pH 5.0) but lower in the remaining soils, varying between 3.7 and 4.4 but with no consistent trend with elevation (Fig.1e).

**Table 3:** Relationships between soil properties and elevation analysed by linear regression (R project). Data illustrated in Figs1 and 2. Non-normal data were square-root- or reciprocal-transformed and checked for normality and homogeneity of variance prior to analysis

	Elevation		
*R*^2^	*F*	*P*	
Total soil C (%)	0.538	75.55	<0.001
Total soil N (%)	0.528	72.68	<0.001
Soil C:N	0.617	104	<0.001
Bulk density of 0–10 cm depth	0.368	39.63	<0.001
Soil pH	0.193	16.1	<0.001
Microbial biomass C	0.457	54.06	<0.001
Microbial biomass N	0.223	19.06	<0.001
Total PLFA	0.651	120.3	<0.001
Fungal PLFA	0.711	158.8	<0.001
Bacterial PLFA	0.559	82.01	<0.001
Gram-positive PLFA	0.510	65.67	<0.001
Gram-negative PLFA	0.581	87.43	<0.001
F:B PLFA	0.703	152.2	<0.001
GP:GN PLFA	0.428	48.96	<0.001

**Figure 2:**
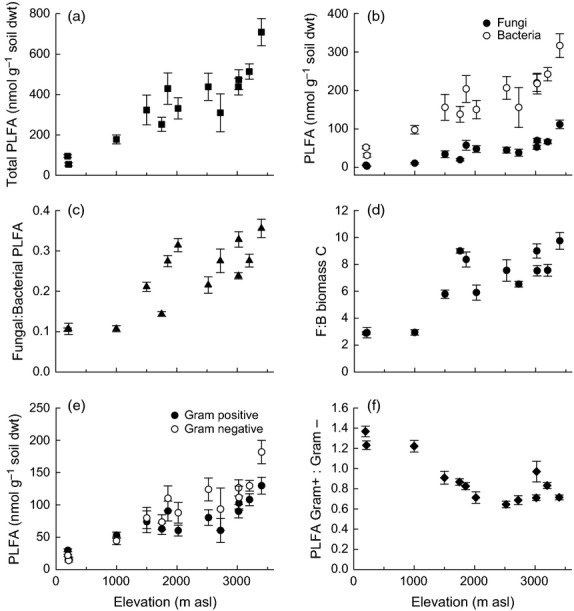
Indicators of microbial biomass and community composition of soils along the elevation gradient: (a) total PLFA, (b) fungal (●) and bacterial (○) PLFA, (c) F:B PLFA, (d) F:B biomass C, (e) gram-positive (●) and gram-negative (○) PLFA and (f) GP:GN PLFA. Data represent mean ± SE (*n* = 5). Statistical analysis is presented in Table3.

Soil biotic properties were also significantly different in the 13 soils, with clear shifts in the size and composition of the microbial community being detected with elevation (Fig.2). Microbial biomass C and N increased significantly with elevation (*P *<* *0.001, *R*^2^ = 0.457, 0.223, respectively), as did the concentration of PLFA biomarkers for total, fungal and bacterial PLFAs (*P *<* *0.001, *R*^2^ = 0.651, 0.711, 0.559, respectively) and gram-positive and gram-negative bacterial PLFAs (*P *<* *0.001, *R*^2^ = 0.510, 0.581; Table3). Total PLFAs increased 10-fold with elevation from 54 to 709 nmol g soil dwt^−1^, whilst fungal and bacterial PLFAs increased from 3.1 to 112 and 31 to 316 nmol g soil dwt^−1^, respectively, from the lowest to highest elevations (Fig.2a,b). The ratio of F:B PLFA also increased significantly from 0.10 to 0.36 (*P *<* *0.001, *R*^2^ = 0.683), which equates to an increase from 2.89 to 9.75 F:B biomass C from the lowest to highest elevation, indicating that fungi are a dominant component of the microbial community at all elevations, with dominance increasing with elevation (Fig.2c,d). In contrast, the ratio of GP:GN PLFA decreased significantly with elevation (*P *<* *0.001, *R*^2^ = 0.428; Table3). Overall these results indicate a clear transition of both soil biotic and abiotic properties upwards along the elevation gradient.

### Experiment 1: Effects of C Quantity and Complexity on Heterotrophic Soil Respiration

**Figure 3:**
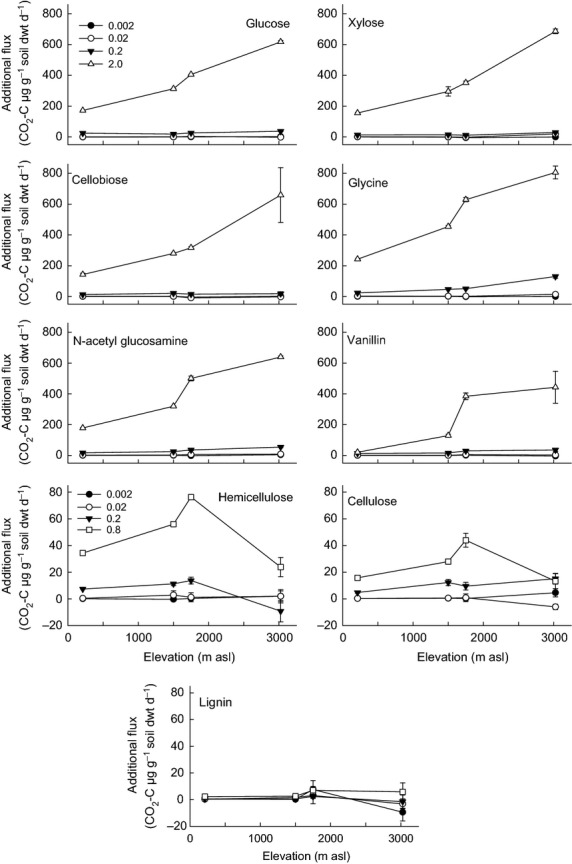
Soil respiration responses to nine C substrates over a range of concentrations (0.002–2.0 mg C g^−1^ soil f. wt.) in tropical forest soils from four elevations. Data represent mean ± SE (*n* = 3) of the additional CO_2_ flux (SIR-BR). SIR, substrate-induced respiration; BR, basal respiration.

Responses of R_H_ to C substrates grouped by their complexity were relatively consistent between soils, with simple compounds generating the greatest additional fluxes (i.e. fluxes after 2 mg C addition: glycine, xylose, cellobiose, glucose, N-acetyl glucosamine > vanillin > hemicellulose, cellulose, lignin; Fig.3). Additional CO_2_ fluxes increased with substrate concentration for most substrate/soil combinations. The exception was lignin, which generated minimal fluxes at all substrate concentrations and in all soils (Fig.3). Comparing the four soils, R_H_ responses to the simple and intermediate C substrates at 2 mg addition were greatest in soils from higher elevations, with a similar trend observed in the 0.2 mg treatments for some simple and intermediate compounds (glycine, N-acetyl glucosamine and vanillin). For the complex substrates, there was no difference in additional CO_2_ flux with elevation, although the response to hemicellulose in the mid-elevation soils (1500 and 1750 m) was greater than in the low- and high-elevation soils (Fig.3). It is likely that the short incubation time used in these experiments limited substrate response to these more complex compounds as reported in other studies (Rinnan & Bååth 2009).

### Experiment 2: Microbial Community Composition and R_H_ Responses to C Inputs

**Figure 4:**
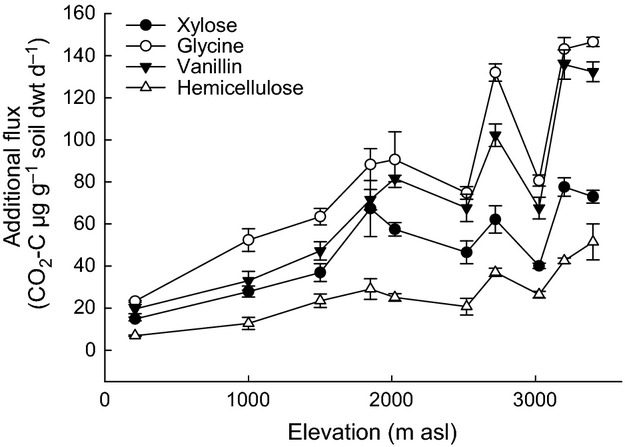
Soil respiration responses to four C substrates in 11 soils from a tropical elevation gradient incubated at 20 °C. Data represent mean ± SE (*n* = 5) of the additional CO_2_ flux (SIR-BR). Two-way anova and Tukey's HSD pairwise comparisons are presented in Table3 and Table S1. SIR, substrate-induced respiration; BR, basal respiration.

Responses of R_H_ to C inputs were measured and compared in 11 soils with distinct abiotic and biotic properties. Basal R_H_, calculated on a mass basis, increased significantly with elevation (*R*^2^ = 0.35, *P *<* *0.001, Fig.1f). There were also significant increases in additional CO_2_ fluxes in response to C inputs in all soil/substrate combinations and also with elevation (*P *<* *0.001, Fig.4 and Table4). The magnitude of R_H_ response to the different substrates followed the same pattern at each elevation along the gradient. Overall, additional CO_2_ fluxes were greatest in response to glycine and vanillin, lower with xylose addition and smallest in response to hemicellulose (Fig.4). The response to C inputs did, however, vary along the elevation gradient, as indicated by the significant soil*substrate interaction (*P *<* *0.0001, Table4). *Post hoc* tests of this interaction demonstrated that additional CO_2_ fluxes in response to hemicellulose were significantly smaller in all soils compared to the other substrates, except for xylose in the 1500 m soil (see Table S1 in Supporting Information). In contrast, CO_2_ fluxes induced by glycine were significantly greater compared to xylose and hemicellulose, except in the 1850 m soil. Further tests of this interaction by substrate revealed that the lowland soil (210 m) was significantly different to all other soils (except the 1000 m soil for xylose, vanillin and hemicellulose) with smaller additional CO_2_ fluxes in response to C inputs (Fig.4 and Table S1). At the upper end of the elevation gradient, however, the two highest elevation soils (3400 and 3200 m) had significantly greater additional CO_2_ fluxes in response to glycine, vanillin and hemicellulose than all other soils below 2720 m elevation (Fig.4 and Table S1).

**Table 4:** Differences in respiration response to substrates of varying complexity in soils from an elevation gradient (Fig.4). Additional CO_2_ flux data (square-root-transformed) analysed by two-way anova with soil and substrate as factors. Pairwise comparisons were performed by Tukey's HSD and are presented in Table S1

Term	d.f.	Sum Sq	Mean Sq	*F* value	*P*
Soil	9	606.0	67.34	124.87	<0.0001
Substrate	3	493.7	164.57	305.20	<0.0001
Soil ^*^ substrate	27	51.6	1.91	3.54	<0.0001
Residuals	159	85.7	0.54		

### Statistical Modelling

To identify the dominant factors driving the observed differences in R_H_ responses to C inputs (Experiment 2), we used statistical modelling to examine the relationships between microbial and abiotic metrics as determinants of R_H_ responses to C inputs (hypothesis 3). We found that in the absence of C inputs, basal R_H_ of soils, incubated at standard temperature and moisture, was best predicted using soil pH, total C and N and their ratio, with these terms explaining most of the attributed variance in the model (Table5). The ratios of F:B and GP:GN PLFAs were retained in the model as non-significant terms, but only explained a small proportion (<2%) of the variance. In contrast, whilst the ‘all substrate’ and individual substrate models used different combinations of soil abiotic and biotic properties in order to best predict R_H_ responses to C inputs, the ratio of F:B PLFAs was consistently included in all the models, explaining between 5.7 and 35.1 % of the attributed model variance (Table5). The response of R_H_ to “all substrates” was best predicted using total and bacterial PLFA, the ratio of F:B PLFAs and soil pH, with total and bacterial PLFAs being the significant terms in the model. The ratio of fungi to bacteria explained most of the attributed variance with a reduction of 35.1% in the explained variance when this term was removed from the model (Table5).

**Table 5:** Linear mixed effects model to determine the relationships between basal (BR) and additional CO_2_ fluxes (SIR-BR) and soil abiotic and biotic properties in 11 soils from the elevation gradient

Term	Control (BR)		All substrates (SIR-BR)		Xylose (SIR-BR)		Glycine (SIR-BR)		Vanillin (SIR-BR)		Hemicellulose (SIR-BR)	
	% Adj. *R*^2^	*P*	% Adj. *R*^2^	*P*	% Adj. *R*^2^	*P*	% Adj. *R*^2^	*P*	% Adj. *R*^2^	*P*	% Adj. *R*^2^	*P*
pH	1.17	*	5.96	ns	1.61	*	0.82	ns	10.63	*	–	–
Total C	4.45	*	–	–	6.04	**	–	–	–	–	3.40	*
Total N	7.36	**	–	–	7.82	ns	10.44	*	–	–	7.55	*
C:N	9.07	**	–	–	–	–	–	–	–	–	–	–
Total PLFA	–	–	3.14	**	–	–	–	–	2.44	*	–	–
Bacterial PLFA	–	–	4.65	**	–	–	–	–	3.90	*	–	–
F:B PLFA	1.34	ns	35.08	ns	15.76	**	5.66	ns	31.68	ns	14.90	**
GP:GN PLFA	0.27	ns	–	–	14.57	ns	11.84	ns	–	–	9.35	*
Total variance explained (Adj *R*[Table-fn tf5-1])	0.813		0.334		0.604		0.652		0.666		0.586	

The relative contribution (%) of each term in explaining model variance was calculated as % difference in adjusted *R*^2^ comparing the full refined model and the model with each term removed. Microbial biomass C and N and fungal PLFA were removed during model refinement.

SIR, substrate-induced respiration; BR, basal respiration.

Symbols indicate the presence or the significance of the term within the refined model: –, not present in refined model; ns, not significant

* =*P *<* *0.05

** = *P *<* *0.01.

Typically, the relative abundance of fungi and bacteria and gram-positive and negative bacteria explained a greater proportion of the model variance than PLFA biomarkers for microbial biomass, or soil abiotic properties. These best-fit models indicate that both biotic and abiotic properties influence R_H_ in these tropical forest soils, but it is the relative abundance of microbial functional groups which has the greatest influence on the magnitude of R_H_ in response to C inputs in soils varying widely in their abiotic properties.

## Discussion

Elevation gradients offer a unique means to examine ecological processes at the landscape and biome scale (Malhi *et al*. 2010). Our analysis showed that soils along an extensive tropical elevation gradient in Andean Peru exhibited a transition in abiotic soil properties which are broadly consistent with reports of increased soil C stocks, C:N ratios and organic layer depth with elevation, in comparable tropical forest elevation gradients in Papua New Guinea, Bolivia and Ecuador (Schawe, Glatzel & Gerold 2007; Wilcke *et al*. 2008; Moser *et al*. 2011; Dieleman *et al*. 2013). Corresponding with these changes in abiotic properties, we also observed differences in the microbial biomass and in the relative abundance of microbial functional groups with elevation (Figs1 and 2). Bacterial and fungal biomass increased with elevation, but the abundance of fungi relative to bacteria also increased, as did the abundance of gram-negative relative to gram-positive bacteria (Fig.2); relationships which have also been observed in Alpine soils and in above-ground fungal communities along this elevation gradient (Margesin *et al*. 2009; Meier *et al*. 2010; Fierer *et al*. 2011).

Taken together, our analyses of soil abiotic and biotic properties illustrate significant changes in the cycling of C and nutrients along the elevation gradient. In the lowlands, microbial biomass, soil C and N contents and litter depth were at the low end of published ranges for tropical soils (Cleveland & Liptzin 2007; Kaspari & Yanoviak 2008), despite high above-ground net primary productivity and warm temperatures promoting high rates of decomposition (Baker *et al*. 2007; Girardin *et al*. 2010; Salinas *et al*. 2011). In these lowland soils, warm temperatures and microbial P limitation (Quesada *et al*. 2010) have likely resulted in low microbial C use efficiency (CUE) with most mineralized C being respired as CO_2_ or invested in extracellular enzymes for P acquisition, rather than being assimilated into microbial biomass (Hartman & Richardson 2013; Waring, Weintraub & Sinsabaugh 2014). Microbial CUE has been shown elsewhere to decrease with increasing temperature and decreasing nutrient availability (Manzoni *et al*. 2012; Frey *et al*. 2013). Also, disproportionate microbial investment in P acquisition has been demonstrated in similar, strongly weathered lowland tropical forest soils (Turner & Wright 2014), whilst low microbial growth efficiency has been correlated with P deficiency in tropical soils (Waring, Weintraub & Sinsabaugh 2014). These studies all support the hypothesis of low microbial CUE in these lowland tropical forest soils. Furthermore, annual respired C along this gradient was estimated to be greater in the lowlands (200 m) compared to montane sites (1000–3030 m; Zimmermann *et al*. 2010), although R_H_ did not change significantly with elevation when measured at five sites from 194 to 3025 m (Girardin *et al*. 2014; Huasco *et al*. 2014; Malhi *et al*. 2014). Given these findings, we hypothesize that in these lowland tropical forest soils, warm temperatures in combination with low P availability have resulted in low microbial CUE through a combination of overflow respiration (greater proportion of C emitted as CO_2_) and greater investment in P acquisition. In contrast, at higher elevations, where temperature and N availability are the main constraints on microbial activity and decomposition, soils with greater microbial biomass and soil C content are present, which contain greater proportions of labile C, which could be mineralized with climate warming (Zimmermann *et al*. 2010, 2012; Salinas *et al*. 2011).

Shifts in the relative abundance of microbial functional groups along the gradient also provide evidence to support our hypothesis (H1) that changes in microbial functional groups correspond with differences in soil C and N status, as a result of differences in C and nutrient availability. Soil bacteria and fungi have differing capacities to degrade available and complex forms of C and N (Nannipieri *et al*. 2003; McGuire *et al*. 2010). Dominance of fungi is often associated with greater recalcitrance of C substrates, as fungi produce extracellular enzymes for the degradation of lignocellulosic material (de Boer *et al*. 2005; Berg & Laskowski 2006). Moreover, bacteria and actinomycetes also produce hydrolytic enzymes for the degradation of cellulose (Berg & Laskowski 2006; Berlemont & Martiny 2013). Nevertheless, given that cellulose in plant material is typically embedded in a matrix of lignin and hemicellulose, it is widely considered that fungi perform the majority of cellulose and lignocellulose degradation, due to their hyphal growth form and enzymatic capacities (de Boer *et al*. 2005). Broad functional groups of bacteria (gram-positive and gram-negative) are also known to differ in their capacity to mineralize C and N (Treseder, Kivlin & Hawkes 2011). Gram-positive bacteria can mineralize recalcitrant organic compounds requiring available inorganic N to invest in extracellular enzymes, whilst gram-negative bacteria target labile C compounds requiring fewer extracellular enzymes and can therefore invest in transport proteins that target organic N (Treseder, Kivlin & Hawkes 2011).

These microbial functional traits fit with our knowledge of nutrient cycling and microbial composition along this gradient. At low elevations (1000 m and below), in soils with low available P and low C:N ratios, the ratio of F:B biomass C was approximately 3 (fungi comprising 75% of microbial biomass), indicating that both bacteria and fungi were playing a significant role in decomposing C inputs, with gram-positive bacteria dominating over gram-negative bacteria. Above 1000 m, the dominance of fungi increased with the ratio of F:B biomass C increasing from 6 to 9.4 (fungi comprising 85–95% of microbial biomass) and gram-negative bacteria more abundant than gram-positive bacteria. These changes corresponded with decreasing litter quality and N availability, and with greater contents of total and labile soil C (Salinas *et al*. 2011; Zimmermann *et al*. 2012), relationships which have also been observed in agricultural and alpine soils (Kramer & Gleixner 2008; Margesin *et al*. 2009; de Vries *et al*. 2012). For example, in an alpine elevation gradient (1000–1900 m asl), the relative amount of fungi to bacteria increased, and gram-positive to gram-negative bacteria decreased with elevation, related to a shift in vegetation from forest to shrubland and grassland (Margesin *et al*. 2009). In addition to C and nutrient availability, temperature also changes significantly along this gradient. Temperature is known to directly affect microbial activity; however, effects on microbial community composition are less clear. Increased dominance of fungi at high elevations in alpine soils was attributed to lower temperatures being more optimal for fungal growth compared with bacteria, which is consistent with our findings, but effects of warmer temperatures on the relative abundance of fungi and bacteria are unknown (Margesin *et al*. 2009). It is therefore likely that there are different and interacting factors controlling the relative abundance of microbial functional groups in these tropical forest soils.

Given these observed differences in biotic and abiotic properties with elevation, we first wanted to test how R_H_ would respond to increasing C input quality and quantity. Through the addition of a wide range of C substrates, we demonstrated that R_H_ increased predictably with increasing C inputs and that increasing inputs of microbially accessible C compounds (glycine and glucose) compared to complex, recalcitrant ones (e.g. lignin and hemicellulose) led to greater relative increases in R_H_ particularly in soils from higher elevations (Fig.3). This supported our second hypothesis that R_H_ responses to C inputs would increase with increasing C quality and quantity. Previous studies examining effects of altered C inputs to soil have reported contradictory, or negligible, effects on soil respiration. For example, the complex substrate phenol was respired significantly less than glucose or glutamate in a coniferous forest soil (Brant, Sulzman & Myrold 2006), whilst in contrast, cellulose addition to a temperate deciduous forest soil resulted in greater respiration rates than glycine and lignin (Hanson *et al*. 2008).

Whilst R_H_ responded predictably to changes in C quality and quantity, there were differences in response amongst soils which corresponded with differences in soil abiotic and biotic properties, including microbial community composition. These differences presumably reflect long-term adaptation to climatic, geological and topographic conditions, which have determined the plant community composition and soil properties at each elevation. In testing our third hypothesis, we found that R_H_ responses to different substrates varied amongst soils with a significant interactive effect between soil and substrate type; for example, the ability to mineralize glycine (simple) and vanillin (intermediate) increased to a greater extent than xylose (simple) or hemicellulose (complex) with increasing elevation (Fig.4 and Table4 and Table S1). Patterns of R_H_ response with elevation span a complex gradient along which many environmental variables change. Increases in R_H_ responses to C inputs corresponded with increases in soil C, N and their ratio, microbial biomass and the relative abundance of fungi to bacteria and gram-positive to gram-negative bacteria (Figs1, 2 and 4). These relationships are not necessarily causal, but indicate that soils with greater C content, C:N ratio and a greater proportion of fungi relative to bacteria had a greater potential to mineralize additional C inputs, consistent with our third hypothesis, that the relative abundance of microbial functional groups would be an important determinant of R_H_ responses to C inputs.

To further test the importance of microbial community composition in influencing R_H_ responses to C inputs, we used a statistical modelling approach (LME). It has been hypothesized that differences in microbial community composition, particularly the relative abundance of fungi and bacteria, are linked to changes in ecosystem C and N cycling (Coleman *et al*. 1978; Bardgett & McAlister 1999; de Vries *et al*. 2012). However, studies relating C substrate mineralization and microbial community composition to C cycling have not been conclusive. For example, soils with different microbial community composition along a plant diversity gradient in the tropical lowlands of Costa Rica varied in their ability to degrade C substrates, with differences in litter decomposition more strongly related to microbial biomass than microbial community composition (Carney & Matson 2005). In another study in Costa Rica, distinct bacterial communities in tropical rain forest soils varied in their ability to decompose a range of C substrates, but showed a consistent capacity to decompose dissolved organic matter substrates of different nutrient quality (Leff *et al*. 2012). Here, we have demonstrated that soils varying widely in abiotic and biotic properties varied in their ability to degrade C substrates, with the composition of the microbial community (F:B and GP:GN PLFA) explaining a greater proportion of the variance (17–35 %) in C substrate mineralization than microbial biomass or soil abiotic properties. This provides evidence to support our third hypothesis that soil microbial community composition is a strong determinant of R_H_ responses to altered C inputs.

Despite the strength of our results, caution is needed when using these findings to predict quantitative effects of changing C inputs on soil respiration in the field. For example, the controlled temperature (20 °C) selected for our experimental incubations lies in the middle of the range of mean annual temperatures observed across the elevation gradient (i.e. ranging from 7.7 to 26 °C; Table1), and there are important differences in soil bulk density and depth along the gradient (Fig.1d). As a result, whilst basal R_H_ increased significantly with elevation on a mass basis (4–130 μg CO_2_-C g^−1^ soil dwt) in our laboratory studies, differences in soil bulk density and *in situ* temperature could account for the observation that field measurements of R_H_ on five of the sites, expressed on an area basis (ha^−1^), did not increase significantly with elevation, ranging from 4.4 to 7.2 Mg C ha^−1^ year^−1^ (Girardin *et al*. 2014; Huasco *et al*. 2014; Malhi *et al*. 2014). Also the effect of these temperature differences on R_H_ along the gradient cannot be predicted using a single Q_10_ function, as the temperature sensitivity of total soil respiration at these sites ranges from 2.1 to 6.9 (Zimmermann *et al*. 2010). In addition, there is evidence that mineralization of substrates of differing complexity also varies in temperature sensitivity, as substrate-specific enzymes have their own characteristic Q_10_ (Frey *et al*. 2013). Nevertheless, we consider that the controlled assays employed in our study can be used to improve understanding of the biotic and abiotic regulators of indirect effects of climate change on soil C cycling along this complex elevation gradient.

Indirect effects of climate change on plant communities are likely to result in changes in the quantity and quality of plant-C inputs. Along this elevation gradient, there is evidence that recent warming (the past ∼30 years) has already caused 2.5–3.5 m year^−1^ upward migration of tree species and it is predicted that for species to remain in equilibrium with their (realized) temperature niche, they will have to migrate upwards by 8 m year^−1^ over the next century (Pinto *et al*. 2009; Feeley *et al*. 2011). Given that microbial composition is a response to both new plant-C inputs and the legacy of existing and historic C inputs, in addition to other soil abiotic properties, microbial communities may migrate, acclimate or adapt in response to changes in plant-C inputs, but the rate at which this happens may differ from that of changes in the plant community (Keiser, Knoepp & Bradford 2013). In the lowland soils (1000 m and below) on this elevation gradient, evidence suggests that the small soil microbial biomass would limit the response to changing C inputs, with nutrient limitation (P) restricting any increase in microbial growth and decomposition rates. We would therefore hypothesize that whilst increases in C quality and quantity in the lowlands may change soil microbial community composition, this would have a negligible effect on R_H_ as P limitation would continue to be the main constraint. The role of microbial CUE, however, needs further examination. As microbial CUE has been shown to decrease with increasing temperature, any warming resulting from climate change could alter the balance of C assimilation and efflux (Frey *et al*. 2013). Consequently, the low microbial CUE of the lowland soils may be further reduced by warming, resulting in a greater proportion of any additional C input being respired as CO_2_. In mid- and high-elevation soils, R_H_ is increasingly limited by N and temperature, which has resulted in the accumulation of significant C stocks known to have a high proportion of labile C (Zimmermann *et al*. 2012). In these soils, we might expect that a combination of increasing C input quality (lower C:N ratio litter) and warming with climate change would decrease N and temperature limitation and result in a shift in microbial composition to communities with relatively fewer fungi and more bacteria. We would hypothesize that changes in microbial community composition combined with reducing N and temperature limitation could lead to significant increases in soil-derived CO_2_ emissions, but the relative contributions of these parameters under a changing climate remain to be quantified. Further research is needed to identify the fate and rate of C mineralization in these soils, and provide improved mechanistic understanding of the effects of nutrient limitation and temperature sensitivity on microbial CUE and soil respiration, in this sensitive region.

## Conclusions

There is growing evidence that climate change is already affecting the elevation range and distribution of trees from the Andes to the western Amazon lowlands (Feeley *et al*. 2011). It is therefore likely that climate change-induced shifts in both soil microbial communities and plant-derived C inputs to the soil will together strongly influence the fate of C reserves in this highly sensitive region. Our results demonstrate that soil microbial community composition, and particularly the relative abundance of fungi and bacteria, is important in determining the response of R_H_ to changes in C inputs in tropical forests. These results also challenge the assumption that different soil microbial communities will be ‘functionally equivalent’ as climate change progresses, which could have important implications for predicted terrestrial C cycle feedbacks (Ostle *et al*. 2009). Greater understanding of plant-mediated effects of climate change on soil microbial communities in tropical soils is needed to improve our predictions of how expected warming will influence soil respiration and overall ecosystem C dynamics.

## Supporting Information

Table S1 Experiment 2: Pairwise comparisons of additional CO_2_ fluxes conducted using Tukey's HSD *post hoc* tests for data subset by soil and substrate and analysed by one-way analysis of variance (All one-way anovas were significant at *P* < 0.0001).
