# Supplementary material for: Microbial community composition explains soil respiration responses to changing carbon inputs along an Andes-to-Amazon elevation gradient
Source: J Ecol. 2014 May 19;102(4):1058–71. doi: 10.1111/1365-2745.12247 (PMC4263258; doi:10.1111/1365-2745.12247)
Supplement: Table S1 — Experiment 2: Pairwise comparisons of additional CO2 fluxes conducted using Tukey's HSD post hoc tests for data subset by soil and substrate and analysed by one-way analysis of variance (All one-way anovas were significant at P < 0.0001). [file jec0102-1058-SD1.docx]

**SUPPORTING INFORMATION**

**Table S1** *Experiment 2:* *Pair-wise comparisons of additional CO_2_ fluxes conducted using Tukeys HSD post-hoc tests for data subset by soil and substrate and analysed by one-way analysis of variance (All one-way ANOVAs were significant at P<0.0001).*

| Substrate | Data subset by soil (elevation m asl) | | | | | | | | | |
| --- | --- | --- | --- | --- | --- | --- | --- | --- | --- | --- |
|  | 210 | 1000 | 1500 | 1850 | 2020 | 2520 | 2720 | 3025 | 3200 | 3400 |
| Xylose | b | b | bc | a | b | b | d | c | c | c |
| Glycine | a | a | a | a | a | a | a | a | a | a |
| Vanillin | ab | b | ab | a | ab | ab | b | a | a | a |
| Hemicellulose | c | c | c | b | c | c | c | b | b | b |

| Elevation  (m asl) | Data subset by substrate | | | |
| --- | --- | --- | --- | --- |
|  | Xylose | Glycine | Vanillin | Hemicellulose |
| 210 | f | d | g | f |
| 1000 | df | c | fg | ef |
| 1500 | cd | bc | cf | cde |
| 1850 | ae | b | b | acd |
| 2020 | abce | b | bd | cde |
| 2520 | bcde | bc | bc | de |
| 2720 | abe | a | de | abc |
| 3025 | bcd | b | bc | acde |
| 3200 | a | a | ae | ab |
| 3400 | a | a | ae | b |
